# Intestinal malrotation as a misdiagnosis of pediatric colchicine resistant familial Mediterranean fever

**DOI:** 10.1186/s12969-015-0044-6

**Published:** 2015-11-10

**Authors:** Merav Heshin-Bekenstein, Philip J. Hashkes

**Affiliations:** Pediatric Department, Shaare Zedek Medical Center, P.O. Box 3235, 9103102 Jerusalem, Israel; Pediatric Rheumatology Unit, Shaare Zedek Medical Center, P.O. Box 3235, 9103102 Jerusalem, Israel

**Keywords:** Familial Mediterranean fever, Intestinal malrotation, Volvulus, Colchicine resistance

## Abstract

**Background:**

Familial Mediterranean fever (FMF) is a disorder characterized by recurrent attacks of fever and serosal inflammation, particularly abdominal pain. Other disease processes, including medical and surgical emergencies, may mimic FMF, especially in atypical cases.

**Case Presentation:**

We present a case of an adolescent male, referred to us with a diagnosis of colchicine resistant FMF, ultimately diagnosed with intestinal malrotation and recurrent volvulus.

**Conclusions:**

In atypical presentations of FMF with potential “red flags”, a thorough patient history is extremely important and should result in prompt referral for the appropriate diagnostic tests.

## Background

Familial Mediterranean Fever (FMF), the most common monogenic autoinflammatory syndrome, is characterized by recurrent episodes of fever and serosal inflammation, including abdominal pain and vomiting. Intestinal malrotation which occurs as a result of an arrest of normal rotation of the embryonic gut, has a varied clinical presentation from infancy through adolescence and adulthood [1, 2]. We present a case of an adolescent male, referred to us with recurrent vomiting and abdominal pain, initially diagnosed with FMF that was resistant to colchicine, ultimately diagnosed with intestinal malrotation.

## Case presentation

**Fig. 1:**
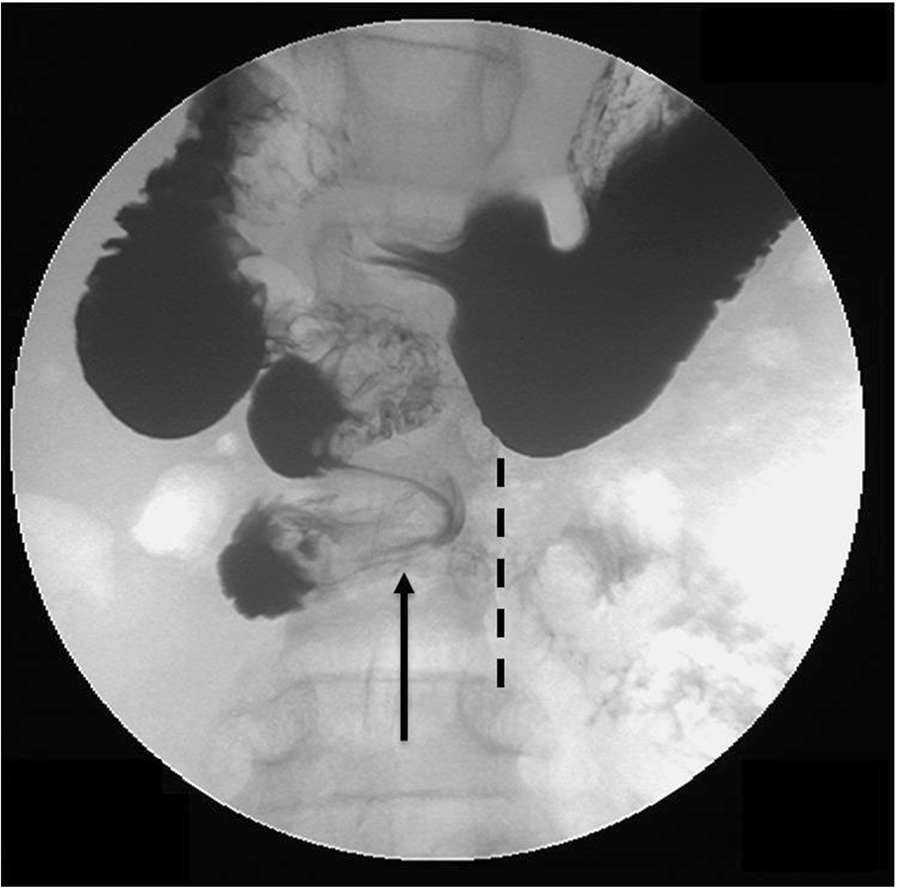
Upper gastrointestinal study (UGI) demonstrating intestinal malrotation and volvulus with abnormal position of the duodenal-jejunal junction to the right of the spine. Normally the duodenum should sweep across from right to left across the spine (dashed line shows the left spinal pedicles). A corkscrew appearance of the duodenum (arrow) demonstrates the volvulus

A 14 year old male of mixed Sephardic-Ashkenazi Jewish ancestry was referred to our Pediatric Rheumatology Clinic with “colchicine resistant FMF”. From early infancy he suffered from recurrent afebrile episodes of yellowish-greenish vomiting with abdominal pain. Episodes occurred every 1 to 2 weeks, lasting for 1–3 days. Exercise, stress and infections were precipitants of episodes. The patient did not develop rashes, joint/muscle or chest pain. He was hospitalized during several episodes due to dehydration, easily corrected by intravenous fluids. His school attendance was severely impaired, missing about 2 days every week. His father was diagnosed with FMF, after years of recurrent fever, abdominal pain and vomiting, with compound heterozygous mutations on the *MEFV* gene (M694V/E148Q). Following father’s diagnosis, our patient underwent genetic analysis at age 11 years and a heterozygote E148Q mutation was found. Colchicine was initiated and he received up to 1.5 mg/d for 2 years prior to his referral, with no clinical improvement. He underwent investigations by multiple pediatric gastroenterologists and metabolic diseases specialists, and his laboratory evaluation was normal including: acute phase reactants (even during episodes), blood counts, liver and muscle enzymes, fibrinogen, celiac profile, amino-acids, organic acids, acyl-carnitine and E3 mutation. Abdominal radiographs and ultrasound were normal. His physical examination was unremarkable except for low weight (7^th^ percentile). Due to the history of bilious vomiting since his neonatal period he was referred by us for an upper gastrointestinal series (UGI). This demonstrated malposition of the ligament of Treitz and a corkscrew appearance of the duodenum, consistent with malrotation (Fig. 1). After a successful Ladd procedure with classical findings of malrotation but no intestinal ischemia, the patient is feeling well, with no further vomiting episodes, a rapid catch up in the growth curves and full school attendance.

## Discussion and conclusion

We present a patient with intestinal malrotation who was initially diagnosed with FMF and treated for 2 years with an adequate dose of colchicine with no response. Fortunately, he did not develop bowel damage after years of recurrent attacks of midgut volvulus.

While the patient fulfilled clinical diagnostic criteria for FMF (Table 1) according to the long-(but not short) version of the Tel-Hashomer criteria [3] (one minor and six supportive criteria), and the Yalcinkaya pediatric criteria [4]**,** several “red-flags” led us to consider an alternative diagnosis.

**Table 1:** Clinical diagnostic criteria for familial Mediterranean fever (FMF)

Tel-Hashomer Criteria (adapted from reference #3: Livneh A, et al. Arthritis Rheum 1997;40:1879–1885)	
Long Version	
Major Criteria	
Typical attacks (≥3 of the same type, rectal temp ≥38 °C, attacks lasting 12 h to 3 days)	
1.Peritonitis	
2.Pleuritis (unilateral) or pericarditis	
3.Monoarthritis (hip, knee, ankle)	
4.Fever alone	
Minor Criteria	
1. Incomplete attacks (typical attacks including one of the following sites: abdomen, chest or joint with 1 or 2 of the following exceptions: 1) Temperature < 38 °C, 2) attacks lasting 6–12 h or 3–7 days, 3) no signs of peritonitis during abdominal attacks, 4) localized abdominal pain, 5) arthritis in joints other than hip, knee or ankle)	
2. Exertional leg pain	
3. Favorable response to colchicine	
Supportive Criteria	
1. Family history of FMF	
2. Appropriate ethnic origin	
3. Age <20 year at disease onset	
4–7 are related to features of attacks:	
4. Severe, requiring bed rest	
5. Spontaneous remission	
6. Symptom-free interval	
7. Transient inflammatory response with one or more abnormal test result(s) for white blood cell count, erythrocyte sedimentation rate, serum amyloid A, and /or fibrinogen	
8. Episodic proteinuria/hematuria	
9. Unproductive laparotomy or removal of “white” appendix	
10. Consanguinity of parents	
An FMF diagnosis requires ≥1 major criteria, or ≥2 minor criteria, or 1 minor criteria plus ≥5 supportive criteria, or 1 minor criteria plus ≥4 of the first 5 supportive criteria.	
Short Version	
Major criteria	
1.Recurrent febrile attacks accompanied by peritonitis, synovitis or pleuritis.	
2.Amyloidosis of the AA-type without predisposing disease.	
3.Favorable response to continuous colchicine treatment.	
Minor criteria	
4. Recurrent febrile attacks	
5. Erysipelas-like erythema	
6. FMF in a first degree relative	
Definitive diagnosis: 2 major or 1 major and 2 minor.	
Probable diagnosis: 1 major and 1 minor	
Yalçinkaya Pediatric Criteria (adapted from reference #4: Yalçinkaya F, et al. Rheumatology (Oxford, England) 2009;48:395–8)	
Criteria	Description
Fever	Axillary temperature of >38 °C, 6–72 h of duration, ≥3 attacks
Abdominal pain	6–72 h of duration, ≥3 attacks
Chest pain	6–72 h of duration, ≥3 attacks
Arthritis	6–72 h of duration, ≥3 attacks, oligoarthritis
Family history of FMF	

Two of five criteria diagnose FMF

1. Neonatal onset of FMF is uncommon [5–7]. About 31 % percent of patients with FMF present by age 2 years (mean 1.1 years) [8], 80 % by 10 years and 90 % by 20 years, but only about 5 % by 2 months of age. The most common presentation in the infants is just “fussiness”. In almost all others fever is part of the presentation [7, 8], thus isolated vomiting in patients under 1 year of age is very uncommon.
2. Bilious vomiting is not characteristic in FMF.
3. The lack of fever and other symptoms characteristic of FMF. These would be expected in a patient with severe FMF based on the frequency of attacks, age of onset and lack of response to colchicine [6, 9].
4. Normal acute phase reactants during attacks.

Besides clinical criteria, the diagnosis of our patient and treatment with colchicine was initially supported by finding a heterozygous E148Q mutation on the *MEFV* gene. Genetic testing in FMF is only considered supportive and not diagnostic, particularly in atypical cases. When two mutations are found, FMF diagnosis is confirmed. In atypical cases where only one mutation is found a trial of colchicine is often offered. Indeed up to 30 % of FMF have only a heterozygote exon ten mutation [10]. However, controversy exists regarding the role of some *MEFV* variations (Table 2), particularly for the exon 2 E148Q glutamic acid to glutamine substitution [11]. Initially, this sequence variation was described as a disease causing mutation with low penetrance and mild symptoms. However, recent studies showed a similar frequency of E148Q among FMF patients and controls, and those findings support the hypothesis that E148Q is a benign polymorphism and not a disease causing mutation [12–14]. E148Q may be a disease modifier in other rheumatologic conditions [15–17]. E148Q may also modify the severity of FMF when found on the same allele with other mutations. FMF patients who are homozygous for the complex allele (E148Q-V726A/E148Q-V726A), or compound heterozygotes (E148Q-V726A/V726A), have a more severe disease compared to patients homozygous for V726A [14]. In our patient the E148Q substitution is likely to be only a polymorphism, unlike his father who had a compound heterozygote M694V/E148Q mutation.

**Table 2:** Clinical significance of the common mutations/variations found in the *MEFV* gene

Mutations associated with classic familial Mediterranean fever (FMF)
M694V
M694I
M680I
V726A
Mutations associated both with classic and atypical FMF
A744S
K695R
R761H
Mutations usually associated with atypical FMF
P369S/R408Q (often in cis)
R329H
Varients considered polymorphisms
^a^E148Q
^a^R202Q

^a^Can be associated with disease in compound heterozygote with other mutations

Multiple other disease processes may closely resemble FMF and must be excluded in atypical cases, such as surgical emergencies, metabolic diseases and other periodic fever syndromes (Table 3) [18, 19]. Both FMF diagnostic criteria have excellent but not 100 % specificity for the population in whom it was developed (99 % for adults with the Tel-Hashomer criteria [3], 92 % for children with the Yalcinkaya criteria [4]). However, the Tel-Hashomer criteria developed for adults had a lower specificity (54 %) in children [4]. Therefore, in atypical cases with potential “red flags” a thorough patient history is extremely important and should result in prompt referral for the appropriate diagnostic tests.

**Table 3:** Differential diagnosis of recurrent vomiting (often with abdominal pain) other than familial Mediterranean fever (FMF) in the pediatric population, by age

Neonate/infancy	Childhood	Adolescence
**GERD**	**GERD**	**GERD**
**Anatomic obstruction** ^a^	>**Anatomic Obstruction** ^a^	**IBD**
Dietary protein intolerance	PUD	**PUD**
Metabolic disorder^b^	Pancreatitis	**Cyclic vomiting**
Renal disorder/obstruction	Cyclic vomiting	Pancreatitis
Adrenal crisis	Metabolic disorders^b^	Biliary colic
		Renal colic
		Acute intermittent porphyria^b^
		Anatomic Obstruction^a^

^a^Includes malrotation with midgut volvulus, pyloric stenosis, intussusception, Hirschsprung disease, congenital atresia/stenosis/webs, incarcerated hernia

^b^Includes urea cycle defects, organic acidemias, fatty acid oxidation defects, disorders of gluconeogenesis in infancy, and porphyria in childhood/adolescence

*GERD* gastroesophageal reflux, *IBD* inflammatory bowel disease, *PUD* peptic ulcer disease

The common causes in each age are marked in **bold.** Functional gastrointestinal disorders are a common cause of recurrent abdominal pain but without vomiting

Malrotation is an incomplete rotation of the intestine during fetal development. The mesentery, including the superior mesenteric artery, is tethered by a narrow stalk, which can twist around itself, and produce life threatening midgut volvulus, with an acute presentation of small bowel obstruction. Traditionally, intestinal malrotation is considered primarily a disease of infancy with infrequent occurrence beyond the first year of life. However, recent studies demonstrate that the prevalence of malrotation in older children and adults appears to be higher than previously thought [1, 2]. Upper gastrointestinal series is the gold standard imaging test for diagnosis. Surgical intervention is recommended regardless of age, and presentation of volvulus requires an emergency procedure.

In summary, alterative diagnoses to FMF should be considered in cases of atypical attacks as in our patient, even if fulfilling diagnostic criteria and supported partially by a heterozygous mutation in the *MEFV* gene. In some cases, as we described, the correct diagnosis may represent a surgical or medical emergency requiring prompt treatment.

## Consent

Written informed consent was obtained from the patient’s parents for publication of this Case Report and any accompanying images. A copy of the written consent is available for review by the Editor-in-Chief of this journal.
